# 3D Printing of PLA/clay Nanocomposites: Influence of Printing Temperature on Printed Samples Properties

**DOI:** 10.3390/ma11101947

**Published:** 2018-10-11

**Authors:** Bartolomeo Coppola, Nicola Cappetti, Luciano Di Maio, Paola Scarfato, Loredana Incarnato

**Affiliations:** Department of Industrial Engineering, University of Salerno, Via Giovanni Paolo II n. 132, 84084 Fisciano (SA), Italy; ncappetti@unisa.it (N.C.); ldimaio@unisa.it (L.D.M.); pscarfato@unisa.it (P.S.); lincarnato@unisa.it (L.I.)

**Keywords:** 3D printing, FDM, nanocomposites, PLA, clay

## Abstract

In this study, the possibility of using a layered silicate-reinforced polylactic acid (PLA) in additive manufacturing applications was investigated. In particular, the aim of this work was to study the influence of printing temperature in the 3D printing process of PLA/clay nanocomposites. For this reason, two PLA grades (4032D and 2003D, D-isomer content 1.5 and 4, respectively) were melt-compounded by a twin screw extruder with a layered silicate (Cloisite 30B) at 4 wt %. Then, PLA and PLA/clay feedstock filaments (diameter 1.75 mm) were produced using a single screw extruder. Dog-bone and prismatic specimens were 3D printed using the FDM technique at three different temperatures, which were progressively increased from melting temperature (185–200–215 °C for PLA 4032D and 165–180–195 °C for PLA 2003D). PLA and PLA/clay specimens were characterized using thermogravimetric analysis (TGA), dynamic mechanical analysis (DMA), differential scanning calorimetry (DSC), and tensile tests. Moreover, the morphology of the 3D printed specimens was investigated using optical microscopy and contact angle measurements. The different polymer matrix and the resulting nanocomposite morphology strongly influenced 3D printed specimen properties. DMA on PLA/clay filaments reported an increase in storage modulus both at ambient temperature and above the glass transition temperature in comparison to neat PLA filaments. Furthermore, the presence of nanoclay increased thermal stability, as demonstrated by TGA, and acted as a nucleating agent, as observed from the DSC measurements. Finally, for 3D printed samples, when increasing printing temperature, a different behavior was observed for the two PLA grades and their nanocomposites. In particular, 3D printed nanocomposite samples exhibited higher elastic modulus than neat PLA specimens, but for PLA 4032D+C30B, elastic modulus increased at increasing printing temperature while for PLA 2003D+C30B slightly decreased. Such different behavior can be explained considering the different polymer macromolecular structure and the different nanocomposite morphology (exfoliated in PLA 4032D matrix and intercalated in PLA 2003D matrix).

## 1. Introduction

Additive manufacturing (AM) technologies are attracting great interest both in the industrial and in the academic fields thanks to several advantages: rapid prototyping, wide choice of materials, and the possibility of realizing objects of complex shapes. Fused Deposition Modeling (FDM) is one of the most investigated AM methods because of its ease of use, low-cost, and that it is usable for processing of traditional thermoplastic polymers (mainly PLA, PS, ABS, Nylon, and PET) [1]. Among these polymers, polylactic acid (PLA) is one of the most used in the FDM process because, by choosing the optimal PLA grade (molecular weight, proportion of D- or L-enantiomers, crystalline morphology, etc.), it is possible to obtain PLA-based polymers with required properties considering the specific application.

PLA is a biodegradable polymer, which is attracting more and more interest in several fields thanks to its good mechanical properties and optical transparency. However, PLA presents several issues such as low thermal stability, crystallization ability, and drawability. A viable strategy to overcome these drawbacks is to reinforce PLA using nanofillers (layered silicates, carbon nanotubes, etc.) [2,3,4,5,6]. The use of filler at the nanoscale allows for simultaneous improvement of both the material’s properties and processability. Drawability and processability are both important in the FDM technology because they influence both feedstock filament production and layer deposition during the printing process. Moreover, the use of nanocomposite materials in the FDM method could overcome one of the main limitations of this technique that is represented by the low mechanical properties of 3D printed parts.

However, although in the last decades several authors investigated the use of polymer/clay nanocomposites for achievable benefits, in particular in terms of the improved mechanical, thermal, and barrier properties [7,8,9,10,11,12], only few researches have been devoted to the use of polymer/clay nanocomposites in the FDM 3D printing [13,14,15,16,17,18,19] and even fewer works have studied the use of PLA/clay nanocomposites [20,21,22,23].

In particular, recent experimental works [22,23] demonstrated the possibility to prepare PLA/clay nanocomposites feedstock filaments that were successfully printed via the FDM technique. 3D printed nanocomposite samples show several advantages, mainly improved shape stability and mechanical properties, with respect to unfilled samples. Moreover, as reported by Cicala et al., the presence of filler in the PLA matrix modifies the polymer matrix rheology, improving the shear thinning behavior and positively influencing the FDM process [24]. Recently, Caminero et al. [25] investigated the 3D printing of continuous fiber reinforced thermoplastic composites reporting promising results in terms of impact resistance. In particular, layer thickness and build orientation were found to be important parameters for 3D printed samples impact strength [25].

Anisotropy and mechanical properties of the printed item are strongly influenced by printing parameters, particularly: printing direction, infill, design of the part, printing speed, and temperature [26,27,28]. Moreover, considering that material fusion is essential in the FDM process, several authors investigated the melting model and the flow during extrusion [29,30,31,32], but, only few authors have studied the influence of 3D printing temperature [33,34]. Yang et al. [33] investigated the effects of printing temperature on polyether ether ketone (PEEK) samples: the higher the printing temperature the higher both tensile strength and elastic modulus. Also, Abbott et al. [34] found that increasing printing temperature resulted in an increase of tensile strength of ABS. The authors explained these results as being due to the increase in polymer coalescence, entanglement, and bond strength among the consecutive layers.

Thus, the aim of this work was to study the influence of 3D printing parameters, in particular printing temperature, on the properties of PLA/clay 3D printed nanocomposites. In particular, two PLAs were used as polymer matrix and a commercial organoclay was used as reinforcing filler. Nanocomposite filaments and 3D printed specimens were characterized using thermogravimetric analysis (TGA), dynamic mechanical analysis (DMA), differential scanning calorimetry (DSC), and tensile tests.

## 2. Materials and Methods

### 2.1. Materials

In this study, two different semicrystalline grades of polylactic acid (PLA) were used as matrix: PLA 4032D and PLA 2003D (NatureWorks, Minnetonka, MN, USA), respectively [35]. The melt flow index (MFI), melting temperature (T_m_), D-isomer content, and polydispersity index (i.e., the ratio between the number average molecular weight, M_n_, and the weight average molecular weight, M_w_), of the two PLA grades are reported in Table 1.

For nanocomposites preparation, an organo-modified layered silicate (Cloisite 30B, Southern Clay Products Inc., Gonzales, TX, USA), modified by methyl, tallow, bis-2-hydroxyethyl, and quaternary ammonium chloride, having a basal interlayer spacing d_001_ = 18.5 Å, was used.

PLA/clay nanocomposites were prepared via melt-compounding using a twin-screw extruder (Dr. Collin GmbH-ZK 25-48D, Dr. Collin GmbH, Ebersberg, Germany) with co-rotating intermeshing screws (D_screw_ = 25 mm, l/d = 42), operating at a screw speed of 200 rpm. During the melt-compounding process, the following temperature profile was used. 160 °C–180 °C–180 °C–180 °C–180 °C–180 °C–180 °C–170 °C (from hopper to die). Prior to processing, PLA pellets and nanoclay powder (4 wt %) were mixed and dried under vacuum (8 h at 80 °C). PLA/clay blends are identified as PLA 4032D+C30B and PLA 2003D+C30B, respectively.

After melt-compounding, both neat PLA and nanocomposite filaments were produced using a single screw extruder (Brabender Do-Corder E330, D_screw_ = 20 mm, L/D = 20) equipped with a capillary die of 3 mm, at a screw speed of 10 rpm, operating at the following temperatures 180–180–160 °C (from hopper to die). Filaments were collected using a take up system with air cooling at 3 m/min in order to have a filament diameter of approximately 1.75 ± 0.10 mm.

These processing conditions allowed to obtain the nanoscale dispersion of C30B organoclay into both PLA matrices, as demonstrated by rheological and TEM analyses reported and discussed in our previous work [2]. In particular, it was found that both the hybrids were characterized by a mixed intercalated/exfoliated nanostructure, and that the size of the intercalated stacks is smaller for the PLA 4032D+C30B than for the PLA 2003D+C30B system, indicating a lower degree of polymer–organoclay interaction than in the last hybrid.

### 2.2. 3D Prinitng

**Figure 1:**
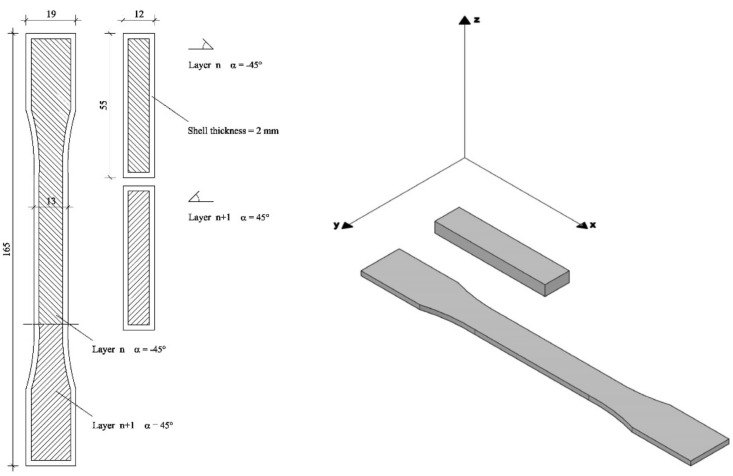
Dog-bone and prismatic specimens dimensions, orientation on the plate, and infill pattern.

PLA and PLA/nanoclay filaments were used to print “dog-bone” specimens, according to the ASTM D638 type I (165 × 19 × 13 mm^3^) and prismatic samples (55 × 12 × 5 mm^3^), previously designed using a CAD software (Figure 1). To print samples, a Sharebot NG (Sharebot S.r.l., Nibionno, Italy) 3D printer was used varying nozzle temperature (progressive increments of 15 °C from melting temperature, as reported in Table 2), but at fixed bed temperature (50 °C), layer height (0.2 mm), and raster angle (+/−45°). Nozzle diameter was 0.35 mm.

The possible defects presence in 3D printed samples was excluded by means of density measurements performed using the Archimedes’ method on three different samples. In all cases, specimens density resulted within the PLA density range (1.24 ± 0.05 g/cm^3^).

### 2.3. Methods

Thermogravimetric analysis (TGA) was carried out on PLA and PLA/clay samples using a Q500 analyzer (TA Instruments, New Castle, DE, USA). Specimens were heated at 10 °C/min from 25 °C to 700 °C under nitrogen atmosphere (20 mL/min).

Differential scanning calorimetry (DSC) was performed on PLA and PLA/clay pellets, filaments and 3D printed samples using a Mettler Toledo Differential Scanning Calorimeter (mod. DSC 822e, Mettler Toledo, Columbus, OH, USA) performing the following thermal cycle under nitrogen atmosphere. First heating at 10 °C/min from 25 °C to 250 °C, then heating an isotherm at 250 °C for 10 min, cooling to 25 °C, and reheating to 250 °C at the same heating rate. During thermal scans, cold crystallization temperature, T_cc_, and melting temperature, T_m_, were recorded. The degree of crystallinity, Χ_c_, of the different samples was calculated by the following equation

(1) $$X\left(\%\right)=\frac{\Delta H_{m}}{\left(1-\phi \right)\Delta H_{100}}\cdot 100,$$

where ΔH_m_ is the heat of crystallization of the sample analyzed (J/g), ΔH_100_ is a reference value that represents the heat of crystallization for a 100% crystalline polymer (for a 100% crystalline PLA is 93 J/g), and ϕ is the organoclay weight percentage.

Tensile tests on printed samples were conducted according ASTM D638 using a universal testing machine (Sans CMT6000 series, Shenzhen, China) equipped with a load cell of 5 kN, at a crosshead speed of 5 mm/min. Mechanical properties are the average of five measurements conducted for each sample and standard deviation values are in the range of 5% to 10%.

Dynamic mechanical thermal properties of PLA and PLA/clay filaments were measured in tensile mode using a DMA Q 800 (TA Instruments, New Castle, DE, USA). Dynamic mechanical testing was conducted using Temp Ramp/Freq Sweep test in Multifrequency Strain method. Testing was carried out in triplicate using an amplitude of 25 μm, frequency of 1 Hz, and preload of 0.01 N in a temperature range of 35 to 100 °C (heating rate of 3 °C/min).

TGA and DSC analysis were performed on three specimens for each sample group, while tensile tests and DMA measurements were carried out on five specimens to ensure reproducibility.

To investigate the influence of printing temperature on dimensional accuracy of PLA and PLA/clay 3D printed samples, an optical microscope (Zeiss Axioskop 40, Carl Zeiss, Oberkochen, Germany) was used.

The influence of printing temperature and nanoclay addition on 3D printed samples surface roughness was analyzed by static contact angle measurements using a contact angle system (FTA 1000 instrument, First Ten Angstroms Inc., Portsmouth, VA, USA) and distilled water as the test liquid. Contact angle pictures were recorded immediately after droplets deposition on the 3D printed specimens; at least five measurements were conducted for each sample.

## 3. Results and Discussion

### 3.1. Thermogravimetric Analysis (TGA)

**Figure 2:**
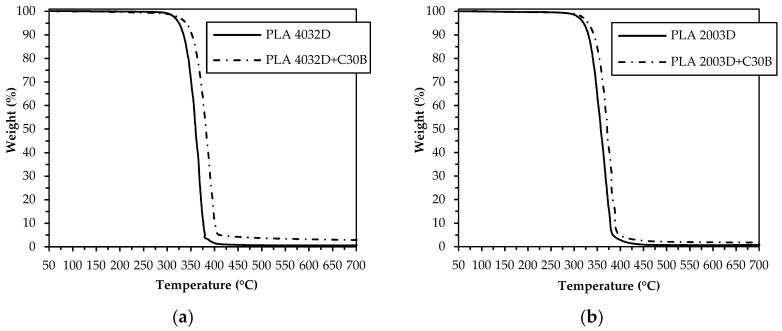
Weight loss of PLA 4032D and PLA 4032D+C30B (**a**) and PLA 2003D and PLA 2003D+C30B (**b**).

In order to evaluate the effect of C30B nanoclay addition on the thermal stability and degradation temperatures of both PLA matrices, TGA analyses were carried out on all neat and nanocomposite PLA samples. Figure 2 compares the thermogravimetric curves and Table 3 reports the temperatures at which samples have a weight loss of 25, 50, and 75% in respect to their initial weight (T_25_, T_50_, and T_75_, respectively). As shown for both PLA nanocomposite systems, the weight loss curve is shifted towards higher temperatures with respect to the corresponding neat matrix. In particular, the increase of thermal stability is more pronounced in the case of PLA 4032D+C30B respect to the other PLA grade (+20 °C and +8 °C, respectively).

Similar beneficial effects have been reported in the literature for several polymer/clay nanocomposites and are generally attributed to the hindering ability of nanodispersed clay platelets towards the transport of gases and volatile degradation molecules across the sample (the better the exfoliation the more effective the hindering effect is), thus affecting the decomposition reactions kinetics [36]. In our case, the higher thermal stability of nanocomposites based on PLA 4032D, compared to those based on PLA 2003D, can be related to their better nanoscale morphology, characterized by a smaller size of the intercalated stacks and a more uniform distribution of both the intercalated stacks and the exfoliated layers [2].

### 3.2. Dynamic Mechanical Thermal Analysis (DMA) of Filaments

**Figure 3:**
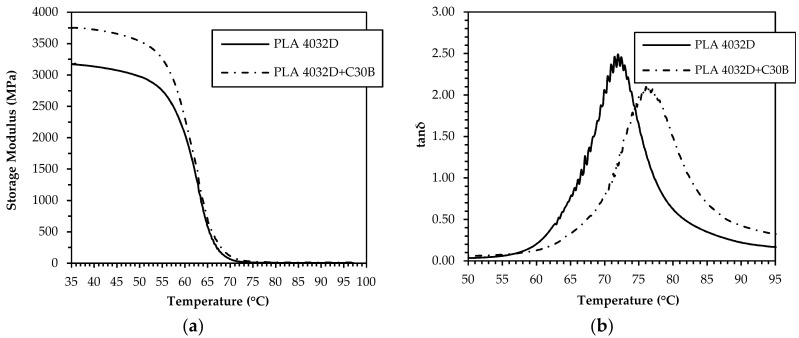
Storage modulus (**a**) and tanδ (**b**) of PLA 4032D and PLA 4032D+C30B filaments.

**Figure 4:**
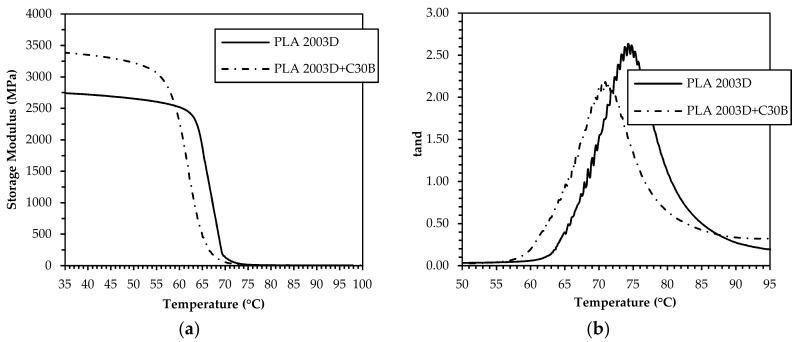
Storage modulus (**a**) and tanδ (**b**) of PLA 2003D and PLA 2003D+C30B filaments.

Neat and nanocomposite filaments produced from both PLA resins were submitted to dynamic mechanical thermal analysis in order to investigate their viscoelastic response and effect on the 3D printing process. The results, in terms of storage modulus and tanδ variation with temperature, are reported in Figure 3 and Figure 4, respectively.

Concerning the PLA 4032D-based samples, Figure 3a shows that the nanocomposite system exhibits a higher storage modulus than the neat matrix in the whole temperature range, which indicates the effectiveness of C30B nanoclay in reinforcing PLA. The difference is remarkable in the glassy state but significantly drops at approximately 60 °C, where the transition from the glassy to rubbery state occurs. Moreover, the tanδ peak (Figure 3b) of PLA 4032D+C30B is shifted towards higher temperatures and has a lower height, thus demonstrating a reduction of the polymer chains mobility as a consequence of the confinement effect caused by the nanodispersed clay. Nevertheless, in the case of PLA 2003D-based systems, even if the nanoclay addition markedly increases the storage modulus in the glassy state, the glass–rubber transition is shifted at lower temperature and thus no stiffness gain in the rubbery plateau region is achieved. This behavior may be related to the existence, in the PLA 2003D+C30B sample, of regions with different densities and higher free volume than the unfilled matrix [37,38], resulting from the presence of quite big stacks of intercalated filler and from the poor polymer chain stereoregularity. However, in this case the tanδ peak height and peak area of the nanocomposite system are lower than that of neat matrix because the elastic component increases more than the viscous component, indicating a decrease in the mobility of the polymer chains.

### 3.3. Differential Scanning Calorimetry (DSC) of Filaments

**Figure 5:**
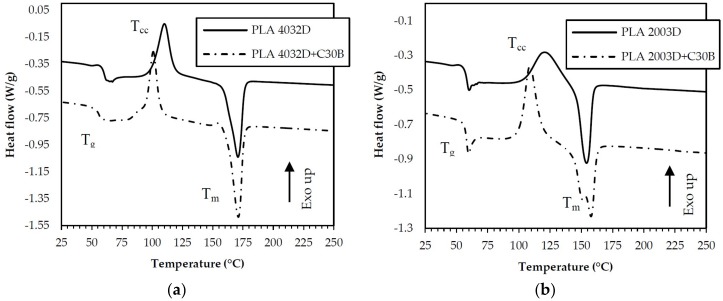
First heating of the filaments of PLA 4032D and PLA 4032D+C30B (**a**) and PLA 2003D and PLA 2003D+C30B (**b**).

With the aim to obtain information on the morphological state and thermal behavior of the investigated systems, DSC measurements were carried out. The obtained thermograms and the corresponding thermal parameters are reported in Figure 5 and Table 5, respectively. The DSC curves show that, for all samples, the glass transition occurred at comparable temperatures, at approximately 60 °C, and is always accompanied by a small endothermic peak, due to enthalpy relaxation of the amorphous glassy materials from nonstable chain conformations towards a more stable state. With respect to the unfilled PLAs, and coherently with the DMA outcomes, this enthalpy change decreases in PLA 4032D+C30B, where the clay restricts the polymer chain mobility, and increases in PLA 2003D+C30B, where the nanoclays involve the growing of free volume. In terms of crystallinity behavior, for both PLAs the nanoclay presence promoted crystallization, decreasing the cold crystallization temperature (T_cc_) of approximately 10 °C (Table 4) and resulting in a narrower and sharper cold crystallization peak. However, the crystallinity degree remains essentially unchanged in all cases, even if, due to the high D-isomer content of PLA 2003D, the crystallization ability of PLA 2003D samples is lower compared to the other PLA grade. Additionally, DSC curves show that there are two melting peaks in PLA 2003D+C30B nanocomposites and only one in the neat PLA 2003D. As stated by several authors and from the findings of our preliminary work [22], the peak at the lower temperature relates to the melting of the disordered α form (α′), while the second peak corresponds to the melting of the α form.

In the cooling thermograms of the investigated samples no crystallization peaks from the melt are recognizable (data not shown). 

### 3.4. Morphology of 3D Printed Samples

**Figure 6:**
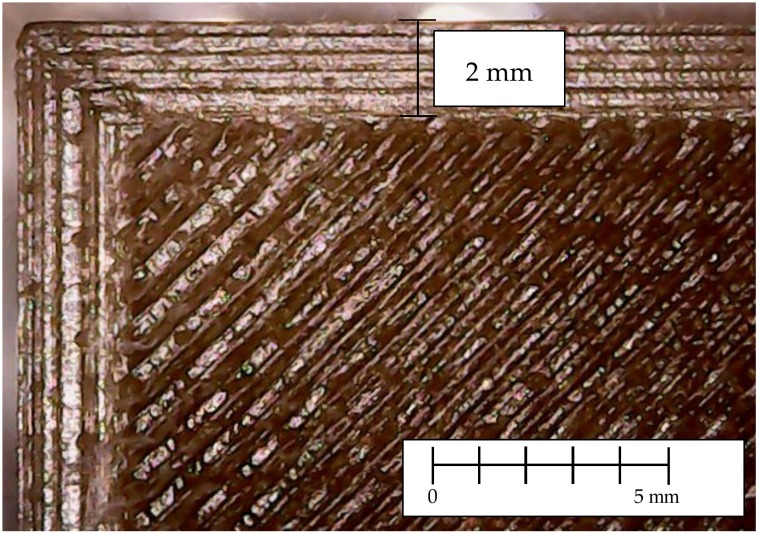
Detail of the outer wall and raster of a 3D printed specimen.

**Figure 7:**
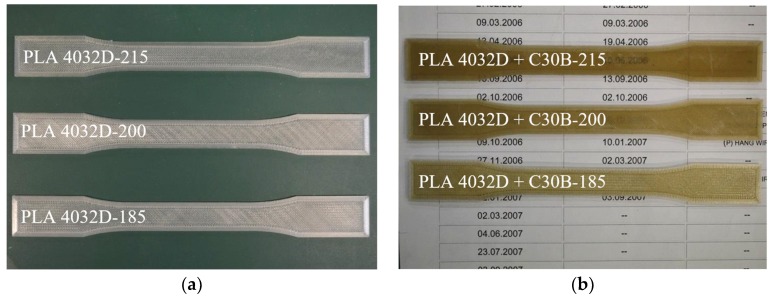
3D printed “dog-bone” specimens: (**a**) PLA4032D and (**b**) PLA4032D+C30B.

The morphology of the 3D printed samples was investigated using an optical microscope and a detail picture of a sample corner is reported in Figure 6. Pictures of “dog-bone” specimens of PLA4032D and PLA4032D+C30B, printed at different temperatures, are reported in Figure 7. As evident, the nanoclay presence changed samples color, resulting in browner specimens. Moreover, both for PLA4032D and PLA4032D+C30B, at increasing 3D printing temperature the specimens become increasingly transparent. The phenomenon can be seen in Figure 7b, where the numbers under the specimens become progressively more readable passing from 185 °C to 215 °C. Such behavior cannot be explained by considering a different degree of crystallinity because, as stated before, all 3D printed samples have approximately the same crystallinity degree that is also close to zero. The different transparency is a result of the different surface roughness (smoother surfaces are more transparent) [39].

**Figure 8:**
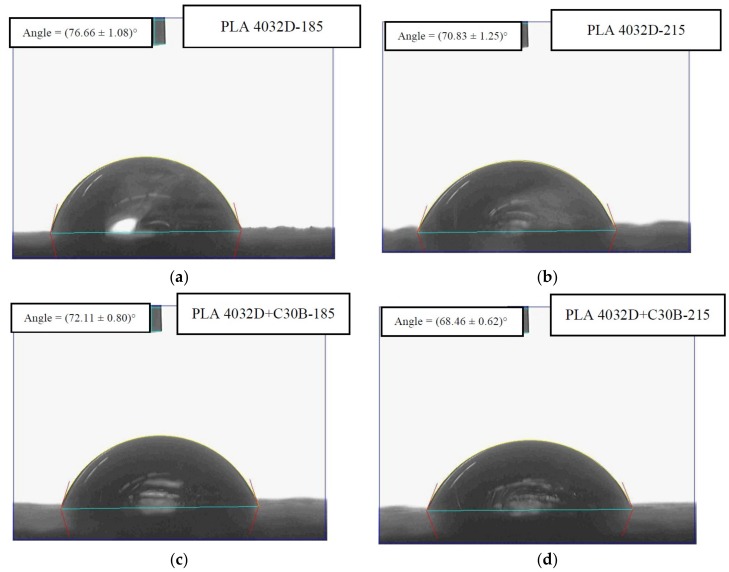
Contact angle of (**a**) PLA 4032D-185, (**b**) PLA 4032D-215, (**c**) PLA 4032D+C30B-185, (**d**) PLA 4032D+C30B-215.

**Figure 9:**
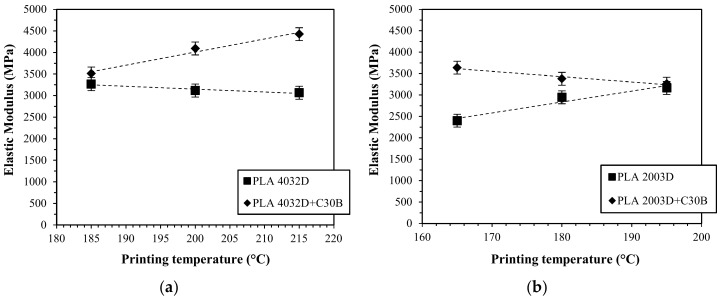
Elastic modulus of the 3D printed samples of PLA 4032D and PLA 4032D+C30B (**a**) and PLA 2003D and PLA 2003D+C30B (**b**) printed at different temperatures.

To investigate specimens’ surface roughness, contact angle measurements were carried out. Contact angles of different 3D printed specimens are reported in Figure 8 and Figure 9. As evident, PLA 4032D and PLA 4032D+C30B specimens printed at the higher temperature (Figure 8b,d, respectively) have a lower contact angle compared to the samples printed at 185 °C (Figure 8a,c, respectively). Moreover, nanocomposite specimens (Figure 8c,d) exhibit lower contact angles compared to neat PLA samples (Figure 8a,b) due to the polar nature of layered silicates. The same considerations can be done also for PLA 2003D and PLA 2003D+C30B.

### 3.5. Tensile Tests of 3D Printed Samples

After filament characterization, PLA and PLA/clay dog-bone specimens were 3D printed, according the different nozzle temperatures reported in Table 2, to perform tensile tests. The elastic modulus (E), elongation at break (ε_b_), and tensile strength (σ) of PLA 4032D, PLA 2003D, and their nanocomposites, printed at different temperatures, are reported in Table 5 and Table 6, respectively.

Coherently to what has been previously observed in terms of degree of crystallinity, PLA 4032D is stiffer than PLA 2003D, but more brittle. In both cases, the nanocomposites have higher elastic modulus compared to pure PLA samples for all the investigated printing temperatures. However, an embrittlement, which increased with nozzle temperature, was observed for both PLA/nanocomposite printed specimens. On the contrary, tensile strength was only slightly affected by clay addition while an influence of the 3D printing temperature was recognizable.

In particular, considering the influence of printing temperature on the elastic modulus of PLA 4032D and PLA 2003D and their nanocomposites, two different behaviors were observed (Figure 9). In the case of PLA 4032D, at increasing printing temperature a slight decrease in elastic modulus was measured (approximately 300 MPa from 185 to 215 °C) while for PLA 4032D+C30B (Figure 9a) at increasing printing temperature elastic modulus increases (approximately 1000 MPa from 185 to 215 °C). On the contrary, in the case of PLA 2003D, at increasing printing temperature an increase of elastic modulus was measured (approximately 800 MPa from 165 to 195 °C) while for PLA 2003D+C30B (Figure 9b) at increasing printing temperature elastic modulus slightly decreases (approximately 350 MPa from 165 to 195 °C).

**Figure 10:**
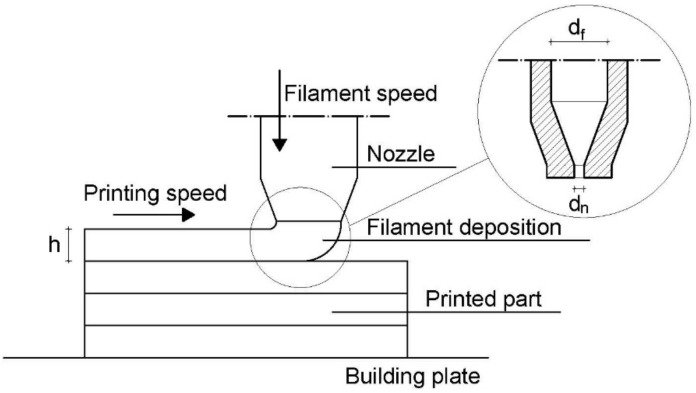
Schematic representation of the filament deposition process and detail of the nozzle.

To explain these differences, attention must be paid to the filament deposition process in which the molten polymer is extruded from the nozzle and deposed on the already printed part (Figure 10). In the printcore, the filament in a semimolten state is pushed through the nozzle at the defined printing temperature. As reported in the literature [16,40,41], the polymer melt undergoes to a pressure drop due to the constrained convergence from the filament diameter (d_f_) to the nozzle diameter (d_n_), resulting in an elongational flow able to orientate both macromolecular chains and layered silicates. Moreover, to a lesser extent, filament stretching occurs also in the semimolten state due to the speed gradient between the filament speed and printing speed (Figure 10). For PLA 4032D, at increasing printing temperature, the polymer matrix is more relaxed avoiding chain orientation and stretching phenomena. On the contrary, in the case of PLA 4032D+C30B, the presence of clay platelets reduces PLA relaxation at the same time allowing the orientation both of layered silicates and the polymer matrix. Thus, as widely known, higher mechanical properties are obtained when polymer and clay orientation occur [7,8].

The opposite behavior was observed for PLA 2003D and PLA 2003D+C30B nanocomposites (Figure 9b). Such different behavior can be explained considering the different polymer architecture and in particular the influence of entanglements. As described in a previous work [2], elongational viscosity curves of PLA 2003D present a strain-hardening behavior under uniaxial extensional flow and it is well-known that at increasing D-isomer content the number of entanglements increases [42]. Moreover, a lower molecular weight between entanglements can promote a strain hardening behavior [43]. Thus, neat PLA 2003D is able to stretch and orient under extensional flow while PLA 2003D+C30B orientation is hindered by layered silicates due to the prevalence of an intercalated morphology. Such assumptions were confirmed by the loss of strain hardening behavior for the PLA 2003D+C30B system reported in a previous work [2].

### 3.6. DMA of 3D Printed Samples

**Figure 11:**
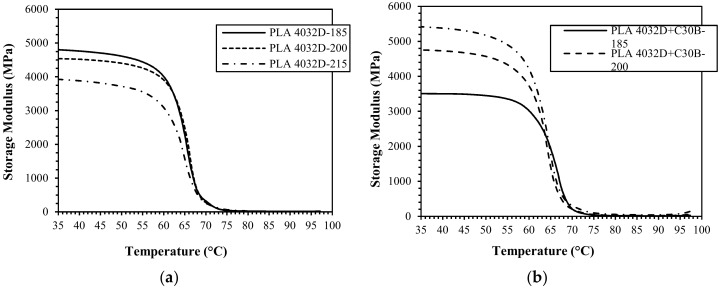

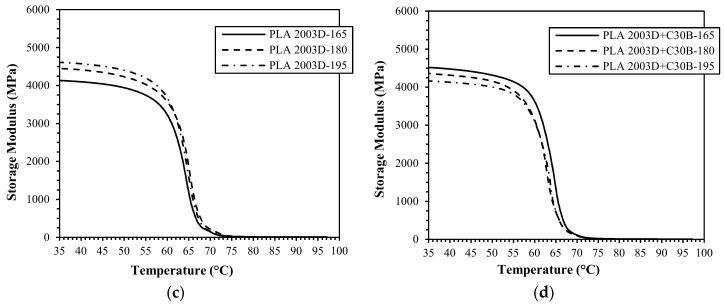
Storage modulus vs. temperature of the 3D printed samples of PLA 4032D (**a**), PLA 4032D+C30B (**b**), PLA 2003D (**c**), and PLA 2003D+C30B (**d**) printed at different temperatures.

Prismatic specimens were 3D printed to investigate viscoelastic properties using DMA in dual-cantilever mode. Storage modulus variation with temperature of 3D-printed samples of neat and nanocomposite PLA 4032D and PLA 2003D, printed at different temperatures, is reported in Figure 11.

Also in this case, accordingly to what was previously discussed, two different behaviors are recognizable for the storage modulus at 35 °C for the two PLA grades. For PLA 4032D storage modulus decreases (approximately 13% from 180 to 215 °C) at increasing printing temperature while for PLA 4032D+C30B a sharp increase of storage modulus (approximately 35% from 180 to 215 °C) was measured at increasing printing temperature. On the contrary, for neat PLA 2003D an increase of storage modulus was obtained at increasing printing temperature (approximately 12% from 165 to 195 °C) while a slight decrease (approximately 4% from 165 to 195 °C) was measured for PLA 2003D+C30B nanocomposites.

The tanδ and storage modulus of PLA and PLA/clay 3D printed specimens at 35 and 80 °C are reported in Table 7. No meaningful tanδ variations were measured at increasing printing temperature and storage modulus variations at 80 °C ($E_{80}^{\prime }$) were negligible. It should be considered that above glass transition temperature, relaxation phenomena occurs and imposed orientations are deleted.

### 3.7. DSC of Printed Samples

To investigate the influence of the 3D printing process on the thermal properties of PLA and PLA nanocomposite samples, DSC analysis was performed on 3D printed specimens. Thermal temperatures and the degree of crystallinity of PLA 4032D, PLA 2003D, and their nanocomposites are reported in Table 8 and Table 9, respectively. Only the results of the first heating are reported in order to correlate such results with the mechanical properties of 3D printed specimens.

Also in this case, accordingly to what was previously observed for neat PLA and PLA/clay filaments, the main differences among the two PLA grades are the different crystallinity degrees and the nucleating effect of the layered silicates. As stated before, due to the different D-isomer content and morphological architecture, PLA 2003D crystallizes with more difficultly compared to PLA 4032D, resulting in a lower *X*_c_ (Table 8 and Table 9). However, both PLAs and their nanocomposites are completely amorphous in the II heating scan due to the rapid cooling process (−10 °C/min). For both PLA/clay nanocomposites, cold crystallization temperature decreases compared to neat PLA matrices, both in the first and in the second heating.

Considering the influence of 3D printing temperature, slight differences are noticeable among thermal parameters of the first heating. Moreover, comparing glass transition temperatures with the corresponding values of the filaments (Table 4), an increase of T_g_ is recognizable for PLA 4032D+C30B and PLA 2003D printed specimens (approximately + 6 and 8%, respectively). Such a variation can be related to the polymer and polymer/clay orientation during the fused filament deposition process. PLA 4032D+C30B and PLA 2003D printed specimens reported an increase of mechanical properties and glass transition temperature, both related to the more ordered morphology obtained. 

**Figure 12:**
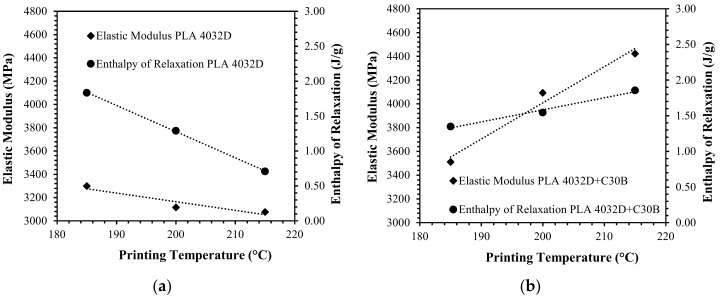
Correlation between elastic modulus and enthalpy of relaxation and 3D printing temperature for PLA 4032D (**a**) and PLA 4032D+C30B (**b**).

**Figure 13:**
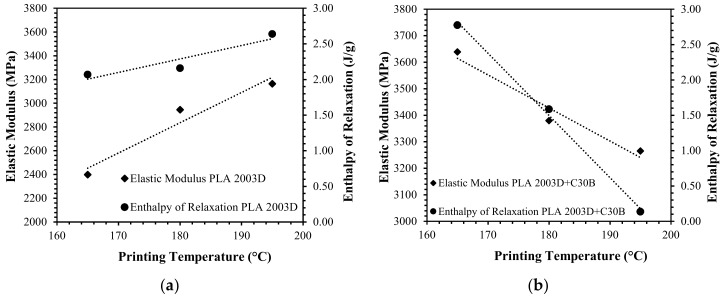
Correlation between elastic modulus and enthalpy of relaxation and 3D printing temperature for PLA 2003D (**a**) and PLA 2003D+C30B (**b**).

The above-mentioned hypothesis produces further confirmation considering the first heating thermograms of PLA 4032D and PLA 2003D printed specimens. In particular, Figure 12 and Figure 13 reports (for 3D printed specimens) the elastic modulus and enthalpy of relaxation considering the different printing temperatures. As is evident, the elastic modulus and enthalpy of relaxation have the same behavior: at increasing enthalpy of relaxation, an increase in elastic modulus is displayed. Once again, the two PLAs and their nanocomposites behave oppositely: for PLA 4032D+C30B and PLA 2003D, at increasing printing temperature an increase in enthalpy of relaxation was measured; on the contrary, for PLA 4032D and PLA 200D+C30B, at increasing printing temperature, enthalpy of relaxation decreases. Such results confirm what has been previously hypothesized regarding orientation phenomena during fused deposition.

## 4. Conclusions

Since printing temperature is one of the most important parameters in the FDM process, in this work, the influence of printing temperature on the properties of two PLA/clay nanocomposites was investigated. Three nozzle temperatures were chosen for each PLA, considering increasing steps of 15 °C in melting temperature. DMA on PLA/clay filaments reported an increase of storage modulus at 35 °C in comparison to neat PLA filaments (+8 and 23% for PLA 4032D+C30B and PLA 2003D+C30B, respectively). Moreover, the nanoclays increased thermal stability, as demonstrated by TGA, and acted as nucleating agent as observed from DSC measurements. The tensile strength of PLA/clay nanocomposites samples obtained by FDM 3D printer at different temperatures was tested. Our results showed that elastic modulus is affected by layered silicates for both PLA matrixes and at all printing temperatures. However, at increasing printing temperature different behaviors were observed for the two PLA grades and their nanocomposites. For PLA 4032D+C30B, elastic modulus increased at increasing printing temperature, whereas it slightly decreased for PLA 2003D+C30B.

The different polymer matrices and the resulting nanocomposite morphologies strongly influenced the properties of the 3D printed specimens. In particular, the different macromolecular architecture of the two matrixes was responsible of the polymer ability to orient or not, as confirmed by 3D printed samples enthalpy of relaxation. Finally, printing temperature also had an influence on 3D printed specimen transparency: the higher the printing temperature the higher the transparency.

This study demonstrated that printing temperature should be chosen considering not only melting temperature, but also polymer architecture and/or nanocomposite morphology in the case of nanocomposite systems. Therefore, potential applications could be found in both considering the improvement in mechanical properties, if the correct temperature is used, and physical/aesthetical properties such as different degree of transparency.

## Figures and Tables

**Table 1:** Melt flow index (MFI), melting temperature (T_m_), D-isomer content, and polydispersity index of the two polylactic acid (PLA) grades.

Polymer	T_m_ (°C)	MFI (g/10 min)	D-isomer (%)	M_w_/M_n_
PLA 4032D	170	5.89	1.5	1.46
PLA 2003D	150	5.92	4	1.46

**Table 2:** 3D printing temperatures for the two PLA grades.

Polymer	T_1_ (°C)	T_2_ (°C)	T_3_ (°C)
PLA 4032D	185	200	215
PLA 2003D	165	180	195

**Table 3:** Temperatures corresponding to a weight loss of 25, 50, and 75% in respect to the initial weight.

Sample	T_25_ (°C)	T_50_ (°C)	T_75_ (°C)
PLA 4032D	348	360	370
PLA 4032D+C30B	368	382	393
PLA 2003D	346	360	372
PLA 2003D+C30B	354	368	379

**Table 4:** Thermal temperatures, enthalpies, and the degree of crystallinity of PLA and PLA/clay filaments.

Heating Scan	Sample	T_g_ (°C)	T_cc_ (°C)	ΔH_cc_ (J/g)	T_m_ (°C)	ΔH_m_ (°C)	*X*_c_ (%)
**First Heating**	PLA 4032D	62	110	31	171	38	8
	PLA 4032D+C30B	58	101	31	171	38	7
	PLA 2003D	60	121	23	154	25	2
	PLA 2003D+C30B	60	109	32	151,158	34	2

**Table 5:** Elastic modulus (E), elongation at break (ε_b_), and tensile strength (σ) of PLA 4032D and PLA 4032D+C30B specimens printed at different temperatures (standard deviation ± 5–10%).

Sample	E (MPa)	ε_b_ (%)	σ (MPa)
PLA 4032D-185	3330	4.04	50
PLA 4032D-200	3117	4.58	49
PLA 4032D-215	3077	5.76	51
PLA 4032D+C30B-185	3511	2.11	40
PLA 4032D+C30B-200	4093	1.85	46
PLA 4032D+C30B-215	4423	1.24	47

**Table 6:** Elastic modulus (E), elongation at break (ε_b_), and tensile strength (σ) of PLA 2003D and PLA 2003D+C30B specimens printed at different temperatures (standard deviation ± 5–10%).

Sample	E (MPa)	ε_b_ (%)	σ (MPa)
PLA 2003D-165	2399	5.56	37
PLA 2003D-180	2945	5.79	47
PLA 2003D-195	3164	6.18	52
PLA 2003D+C30B-165	3639	5.87	52
PLA 2003D+C30B-180	3379	3.66	44
PLA 2003D+C30B-195	3264	3.21	43

**Table 7:** Tanδ and storage modulus (dual cantilever mode) of PLA and PLA/clay 3D printed specimens at 35 and 80 °C.

Sample	E′ (MPa)		tanδ Peak (°C)
	35 °C	80 °C	
PLA 4032D-185	4671 ± 185	23 ± 1	74 ± 1
PLA 4032D-200	4413 ± 178	19 ± 3	75 ± 2
PLA 4032D-215	4081 ± 221	21 ± 8	76 ± 1
PLA 4032D+C30B-185	3974 ± 225	16 ± 2	74 ± 1
PLA 4032D+C30B-200	4281 ± 172	20 ± 5	74 ± 2
PLA 4032D+C30B-215	5331 ± 85	29 ± 9	73 ± 1
PLA 2003D-165	4104 ± 42	13 ± 1	72 ± 2
PLA 2003D-180	4501 ± 67	14 ± 3	74 ± 1
PLA 2003D-195	4600 ± 16	11 ± 8	73 ± 1
PLA 2003D+C30B-165	4257 ± 271	11 ± 2	72 ± 1
PLA 2003D+C30B-180	4219 ± 200	11 ± 5	72 ± 1
PLA 2003D+C30B-195	4110 ± 75	10 ± 9	72 ± 2

**Table 8:** Thermal temperatures, enthalpies and degree of crystallinity of PLA 4032D and PLA 4032D+C30B printed specimens (first heating).

Sample	T_g_ (°C)	T_cc_ (°C)	T_m_ (°C)	*X*_c_ (%)
PLA 4032D-185	65	110	166,170	3
PLA 4032D-200	64	113	169,172	2
PLA 4032D-215	62	112	168,172	2
PLA 4032D+C30B-185	61	100	170	1
PLA 4032D+C30B-200	62	99	171	3
PLA 4032D+C30B-215	61	100	172	3

**Table 9:** Thermal temperatures, enthalpies and degree of crystallinity of PLA 2003D and PLA 2003D+C30B printed specimens (first heating).

Sample	T_g_ (°C)	T_cc_ (°C)	T_m_ (°C)	*X*_c_ (%)
PLA 2003D-165	66	122	156	1
PLA 2003D-180	65	119	152	1
PLA 2003D-195	64	116	151	1
PLA 2003D+C30B-165	62	108	151,157	1
PLA 2003D+C30B-180	61	107	152,158	1
PLA 2003D+C30B-195	61	108	151,157	1

## References

[1] Mohan N., Senthil P., Vinodh S., Jayanth N. (2017). A review on composite materials and process parameters optimisation for the fused deposition modelling process. Virtual Phys. Prototyp..

[2] Di Maio L., Garofalo E., Scarfato P., Incarnato L. (2015). Effect of polymer/organoclay composition on morphology and rheological properties of polylactide nanocomposites. Polym. Compos..

[3] Di Maio L., Scarfato P., Milana M.R., Feliciani R., Denaro M., Padula G., Incarnato L. (2014). Bionanocomposite polylactic acid/organoclay films: Functional properties and measurement of total and lactic acid specific migration. Packag. Technol. Sci..

[4] Scarfato P., Di Maio L., Incarnato L. (2015). Recent advances and migration issues in biodegradable polymers from renewable sources for food packaging. J. Appl. Polym. Sci..

[5] La Mantia F.P., Arrigo R., Morreale M. (2014). Effect of the orientation and rheological behavior of biodegradable polymer nanocomposites. Eur. Polym. J..

[6] Scaffaro R., Sutera F., Mistretta M.C., Botta L., La Mantia F.P. (2017). Structure-properties relationships in melt reprocessed PLA/hydrotalcites nanocomposites. Express Polym. Lett..

[7] Coppola B., Scarfato P., Incarnato L., Di Maio L. (2017). Morphology development and mechanical properties variation during cold-drawing of polyethylene-clay nanocomposite fibers. Polymers.

[8] La Mantia F.P., Ceraulo M., Mistretta M.C., Botta L. (2018). Effect of the elongational flow on morphology and properties of polypropylene/graphene nanoplatelets nanocomposites. Polym. Test..

[9] Scarfato P., Incarnato L., Di Maio L., Dittrich B., Schartel B. (2016). Influence of a novel organo-silylated clay on the morphology, thermal and burning behavior of low density polyethylene composites. Compos. B Eng..

[10] Garofalo E., Di Maio L., Scarfato P., Di Gregorio F., Incarnato L. (2018). Reactive compatibilization and melt compounding with nanosilicates of post-consumer flexible plastic packagings. Polym. Degrad. Stab..

[11] Garofalo E., Scarfato P., Di Maio L., Incarnato L. (2017). Tuning of co-extrusion processing conditions and film layout to optimize the performances of PA/PE multilayer nanocomposite films for food packaging. Polym. Compos..

[12] Botta L., Scaffaro R., Sutera F., Mistretta M.C. (2017). Reprocessing of PLA/Graphene Nanoplatelets Nanocomposites. Polymers.

[13] Dul S., Fambri L., Pegoretti A. (2016). Fused deposition modelling with ABS–graphene nanocomposites. Composites Part A.

[14] Weng Z., Wang J., Senthil T., Wu L. (2016). Mechanical and thermal properties of ABS/montmorillonite nanocomposites for fused deposition modeling 3D printing. Mater. Des..

[15] Meng S., He H., Jia Y., Yu P., Huang B., Chen J. (2017). Effect of nanoparticles on the mechanical properties of acrylonitrile–butadiene–styrene specimens fabricated by fused deposition modeling. J. Appl. Polym. Sci..

[16] Brenken B., Barocio E., Favaloro A., Kunc V., Pipes R.B. (2018). Fused filament fabrication of fiber-reinforced polymers: A review. Addit. Manuf..

[17] De Leon A.C., Chen Q., Palaganas N.B., Palaganas J.O., Manapat J., Advincula R.C. (2016). High performance polymer nanocomposites for additive manufacturing applications. React. Funct. Polym..

[18] Blok L.G., Longana M.L., Yu H., Woods B.K.S. (2018). An investigation into 3D printing of fibre reinforced thermoplastic composites. Addit. Manuf..

[19] Jiang D., Smith D.E. (2017). Anisotropic mechanical properties of oriented carbon fiber filled polymer composites produced with fused filament fabrication. Addit. Manuf..

[20] Francis V., Jain P.K. (2016). Experimental investigations on fused deposition modelling of polymer-layered silicate nanocomposite. Virtual Phys. Prototyp..

[21] Paspali A., Bao Y., Gawne D.T., Piestert F., Reinelt S. (2018). The influence of nanostructure on the mechanical properties of 3D printed polylactide/nanoclay composites. Compos. B Eng..

[22] Coppola B., Cappetti N., Di Maio L., Scarfato P., Incarnato L. Layered silicate reinforced polylactic acid filaments for 3D printing of polymer nanocomposites. Proceedings of the IEEE 3rd International Forum on Research and Technologies for Society and Industry (RTSI).

[23] Coppola B., Cappetti N., Di Maio L., Scarfato P., Incarnato L. (2018). Influence of 3D printing parameters on the properties of PLA/clay nanocomposites. AIP Conf. Proc..

[24] Cicala G., Giordano D., Tosto C., Filippone G., Recca A., Blanco I. (2018). Polylactide (PLA) filaments a biobased solution for additive manufacturing: Correlating rheology and thermomechanical properties with printing quality. Materials.

[25] Caminero M.A., Chacón J.M., Garcia-Moreno I., Rodriguez G.P. (2018). Impact damage resistance of 3D printed continuous fibre reinforced thermoplastic composites using fused deposition modelling. Compos. B Eng..

[26] Dizon J.R.C., Espera A.H., Chen Q., Advincula R.C. (2017). Mechanical characterization of 3D-printed polymers. Addit. Manuf..

[27] Song Y., Li Y., Song W., Yee K., Lee K.Y., Tagarielli V.L. (2017). Measurements of the mechanical response of unidirectional 3D-printed PLA. Mater. Des..

[28] Chacón J.M., Caminero M.A., García-Plaza E., Núñez P.J. (2017). Additive manufacturing of PLA structures using fused deposition modelling: Effect of process parameters on mechanical properties and their optimal selection. Mater. Des..

[29] McIlroy C., Olmsted P.D. (2017). Disentanglement effects on welding behavior of polymer melts during the fused-filament-fabrication method for additive manufacturing. Polymer.

[30] Peng F., Vogt B.D., Cakmak M. (2018). Complex flow and temperature history during melt extrusion in material extrusion additive manufacturing. Addit. Manuf..

[31] Comminal R., Serdeczny M.P., Pedersen D.B., Spangenberg J. (2018). Numerical modeling of the strand deposition flow in extrusion-based additive manufacturing. Addit. Manuf..

[32] Osswald T.A., Puentes J., Kattinger J. (2018). Fused filament fabrication melting model. Addit. Manuf..

[33] Yang C., Tian X., Li D., Cao Y., Zhao F., Shi C. (2017). Influence of thermal processing conditions in 3D printing on the crystallinity and mechanical properties of PEEK material. J. Mater. Process. Technol..

[34] Abbott A.C., Tandon G.P., Bradford R.L., Koerner H., Baur J.W. (2018). Process-structure-property effects on ABS bond strength in fused filament fabrication. Addit. Manuf..

[35] NatureWorks Home Page. https://www.natureworksllc.com.

[36] Darie R.N., Pâslaru E., Sdrobis A., Pricope G.M., Hitruc G.E., Poiată A., Baklavaridis A., Vasile C. (2014). Effect of nanoclay hydrophilicity on the poly (lactic acid)/clay nanocomposites properties. Ind. Eng. Chem. Res..

[37] Incarnato L., Scarfato P., Russo G.M., Di Maio L., Iannelli P., Acierno D. (2003). Preparation and characterization of new melt compounded copolyamide nanocomposites. Polymer.

[38] Incarnato L., Scarfato P., Scatteia L., Acierno D. (2004). Rheological behavior of new melt compounded copolyamide nanocomposites. Polymer.

[39] Cho K.L., Liaw I.I., Wu A.H.F., Lamb R.N. (2010). Influence of roughness on a transparent superhydrophobic coating. J. Phys. Chem. C.

[40] Turner B.N., Strong R., Gold S.A. (2014). A review of melt extrusion additive manufacturing processes: I. Process design and modeling. Rapid Prototyp. J..

[41] McIlroy C., Olmsted P.D. (2017). Deformation of an amorphous polymer during the fused-filament-fabrication method for additive manufacturing. J. Rheol..

[42] Arias V., Höglund A., Odelius K., Albertsson A.C. (2013). Polylactides with “green” plasticizers: Influence of isomer composition. J. Appl. Polym. Sci..

[43] Sun H., Wang S.Q. (2012). Shear and extensional rheology of entangled polymer melts: Similarities and differences. Sci. China Chem..

